# Comparison of periprosthetic bone remodeling after implantation of anatomic and tapered cementless femoral stems in total hip arthroplasty: A prospective cohort study protocol

**DOI:** 10.1097/MD.0000000000012560

**Published:** 2018-09-28

**Authors:** Xiang-Dong Wu, Yu Chen, Zhang-Yu Wang, Yu-Jian Li, Zheng-Lin Zhu, Yu-Zhang Tao, Hong Chen, Qiang Cheng, Wei Huang

**Affiliations:** Department of Orthopaedic Surgery, The First Affiliated Hospital of Chongqing Medical University, Chongqing, China.

**Keywords:** bone mineral density, bone remodeling, cementless stem, hip arthroplasty, LCU stem, Ribbed stem

## Abstract

**Introduction:**

Current total hip arthroplasty (THA) implant usage trends favor cementless fixation, and plenty studies have demonstrated that numbers of cementless femoral stems are associated with excellent long-term survivorship and functional outcomes. Various types of cementless femoral stems have been developed and utilized in multiple applications, including straight, tapered, anatomic, customized, short, and even neck stems. All of these designs aimed to achieve maximal primary stability and promote osseointegration. Nevertheless, stress-shielding and periprosthetic bone loss continue to occur and remain critical issues in promoting long-term survivorship of THA. Considering anatomic and tapered stems are the most popular cementless designs today, this prospective cohort study aimed to investigate the effect of stem design on stress-shielding and periprosthetic bone remodeling after implantation of an anatomic stem with proximal fixation (Ribbed Hip system; Waldemar Link, Hamburg, Germany) and the direct comparison to a fully coated tapered stem (LCU Hip system; Waldemar Link).

**Materials and methods:**

This prospective cohort study will comprise patients who receive primary unilateral THA with the Ribbed anatomic hydroxyapatite (HA)-coated stem or LCU tapered fully HA-coated stem. The changes in periprosthetic bone mineral density after insertion of Ribbed and LCU stem prostheses will be assessed by means of dual-energy X-ray absorptiometry in the periprosthetic region of interest according to Gruen and colleagues. Standard anteroposterior and lateral plain radiography will be performed for qualitative assessment of the periprosthetic bone remodeling. The following items will be analyzed or measured on follow-up radiographs to compare with the initial appearance on the radiographs taken immediately postoperatively: cortical thickness in each Gruen zone, fitness of the distal stem within the isthmus, femoral stem alignment, radiolucent line, reactive line, periosteal bone reactions, and subsidence. Biologic fixation and stability of the cementless implant will be evaluated using Engh grading scale, and heterotopic ossification will be graded according to Brooker classification. Furthermore, Harris hip score and Western Ontario and McMaster Universities Osteoarthritis Index Score will also be assessed for postoperative functional evaluation. These radiologic and clinical assessments will be taken postoperatively, at 6 months, 1, 2, 3, 4, and 5 years after surgery.

**Ethics and dissemination:**

This study was approved by The First Affiliated Hospital of Chongqing Medical University Ethics Committee. The study results will be disseminated at national and international conferences and published in peer-reviewed journals.

**Study registration:**

Chinese Clinical Trial Registry (http://www.chictr.org.cn): ChiCTR1800017841.

## 1 Introduction

During the past decade, a growing body of evidence supports the use of cementless femoral stems with many studies indicating excellent track record that includes high long-term survival rate and satisfying functional outcomes.^[1–6]^ Therefore, cementless femoral prostheses have become the predominant stem utilized in the United States.^[7]^ However, along with the global trends in population aging and longer life expectancy, and increasing number of total hip arthroplasty (THA) is performed in younger, healthier, and more active patients, the expectations regarding THA are continuously rising, in particular regarding the high durability and long-term survival.^[8–13]^ Clinical longevity of cementless implants mainly depends on the osseointegration; however aseptic loosening, the clinical endpoint of bone resorption around the implant, remains the principle cause of implant failure.^[14–16]^ Stress shielding, an unavoidable mechanical phenomenon after insertion of the femoral stem into the intramedullary canal, has been identified as the primary factor contributing to adaptive periprosthetic bone remodeling and resorption around hip stems.^[17–20]^ Stem geometry of the implant plays a crucial role in the load transfer to the femur and consequently bone adaption, thus optimization of geometry could reduce stress shielding, minimize bone atrophy, and promote long-term survivorship.^[21,22]^

Currently, various types of cementless femoral stems have been developed and utilized in multiple applications, and according to the basic design concepts of cementless femoral stems, the main rationales in stem geometry can be initially classified into 3 types: anatomic designs, straight designs, and tapered designs.^[23–25]^ Recently, customized stems, short stems, and neck stems have been developed to reduce stress shielding and improve long-term stability.^[26,27]^ Each type of design has distinct geometries and philosophies, accompanied with a unique model of load transfer and stress-shielding, which would induce implant-specific periprosthetic bone remodeling.^[28,29]^ Therefore, prospective long-term follow-up of the periprosthetic bone mineral density (BMD) changes would help to evaluate the stress shielding, understand the periprosthetic bone remodeling, which is critical for improving the design of implants, predicting periprosthetic fracture or loosening, and aiding in clinical decisions.

Considering anatomic stems and tapered stems are the most popular cementless designs in primary THA today, and comparison of periprosthetic bone remodeling after implantation of anatomic and tapered cementless femoral stems in THA is relatively limited, we thus designed this prospective cohort study, which aimed to investigate the effect of stem geometry on stress-shielding and periprosthetic bone remodeling after implantation of an anatomic stem with proximal fixation (Ribbed Hip system; Waldemar Link, Hamburg, Germany) and the direct comparison to a fully coated tapered stem (LCU Hip system; Waldemar Link).^[30–33]^

## 2 Materials and methods

This study will be performed and reported in accordance with the STrengthening the Reporting of OBservational studies in Epidemiology (STROBE) checklist.^[34]^

### 2.1 Study design

This is a 5-year prospective longitudinal cohort study, which will be conducted in The First Affiliated Hospital of Chongqing Medical University. It will start recruiting patients in September 2018 and is being prepared now. The study will be executed in 2 phases. Phase I is a cross-sectional study, which will be conduct during hospitalization to obtain the baseline data; phase II comprises a cohort follow-up study at 6 months, 1, 2, 3, 4, and 5 years after THA. Participants will be followed for at least 5 years, or until death.

### 2.2 Participants and eligibility criteria

Adult patients who undergone primary unilateral THA with the Ribbed (Ribbed Hip system; Waldemar Link) cementless anatomic hydroxyapatite (HA) coated femoral stem or LCU (LCU Hip system; Waldemar Link) cementless tapered fully HA-coated femoral stem will be potential eligible for this study. However, subjects who meet any of the following criteria will be excluded from the study: with abnormal deviations of the femoral neck (varus caput-collum-diaphysea [CCD] angle <115°, valgus CCD angle >150°) or other femoral deformities; diagnosed with intertrochanteric fractures or pathologic fractures; or diagnosed with bone tumor, glucocorticoid, hyperthyroidism, hypothyroidism, or any other diseases that affecting bone metabolism. Furthermore, subjects who opt to terminate participation or want to withdraw from the research will be discontinued from the study. The participant who has been withdrawn will be replaced by a new participant if time permits.

### 2.3 Surgical procedures

All surgeries are routinely performed by 2 senior surgeons using posterior-lateral approach under general anesthesia in laminar air flow operation room. Surgeons conduct the preoperative design on the equal proportion digital radiography according to the prosthesis template to predict implant type and size. During the operation phase, the femoral neck will be fully exposed, and the femoral head will be removed by osteotomy under the condition of retaining moderate femoral calcar. Acetabular preparation and implantation of a cementless cup followed standard procedures. Then, the assistant will adduct and internally rotate the hip joint to reveal the end of the femoral neck, and the medullary cavity will be enlarged with reamers of increasing sizes, and the appropriate type of components model will be selected and installed according to the size of it. A definite implant matching the size will be inserted using a handle. Perioperative intravenous of antibiotic cefuroxime and postoperative rivaroxaban are routinely used in prevent of infection and thrombosis provided that no contraindications existed. Intravenous combined with topical tranexamic acid are sequentially used to reduce blood loss and transfusions. Patients will be mobilized using standard physiotherapy program, and immediate full weight-bearing with crutches will be encouraged from the 1st postoperative day.

### 2.4 Implant

**Figure 1:**
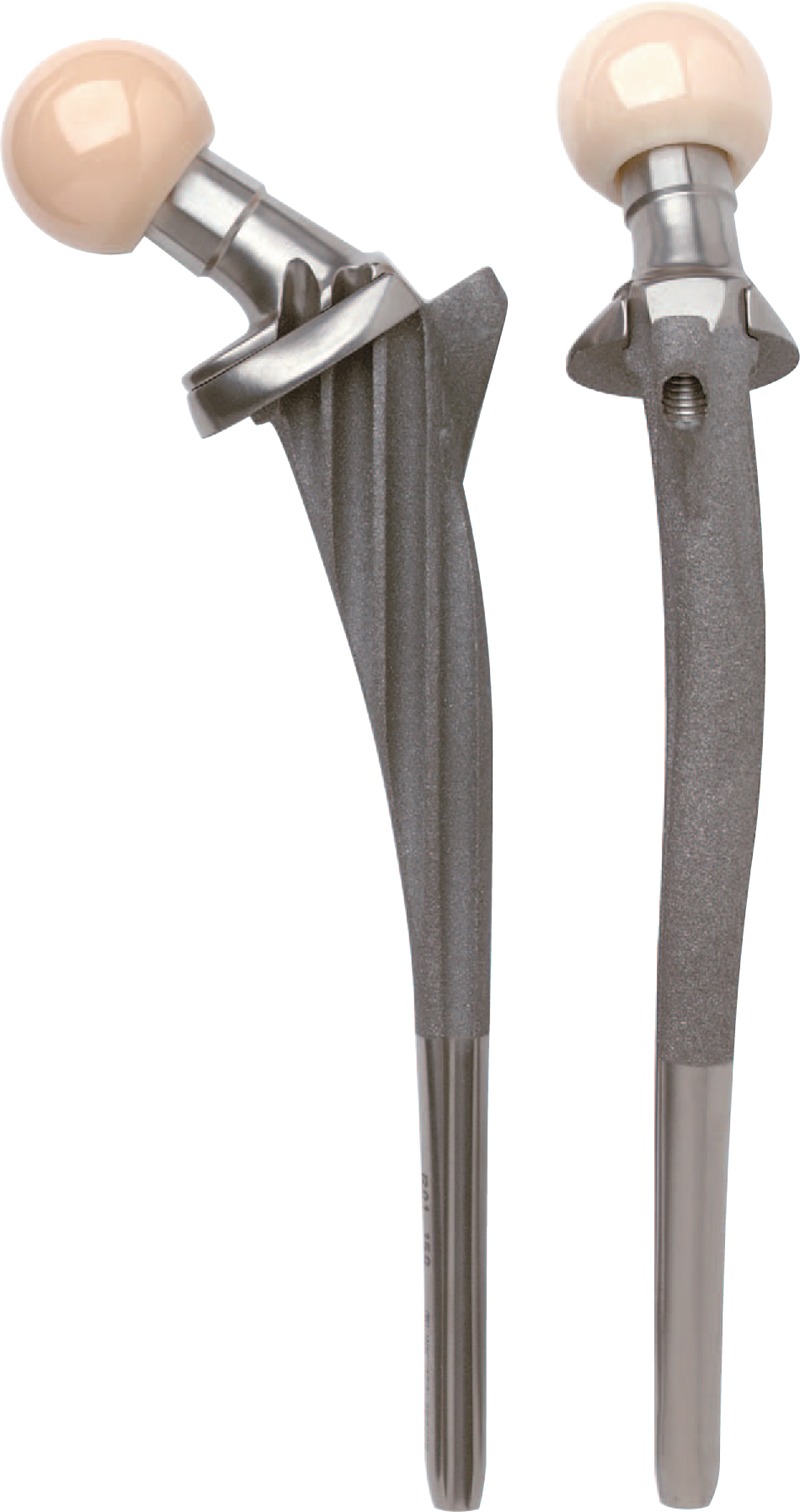
Ribbed cementless anatomic femoral stem (Ribbed Hip system, Waldemar Link, Hamburg, Germany).

**Figure 2:**
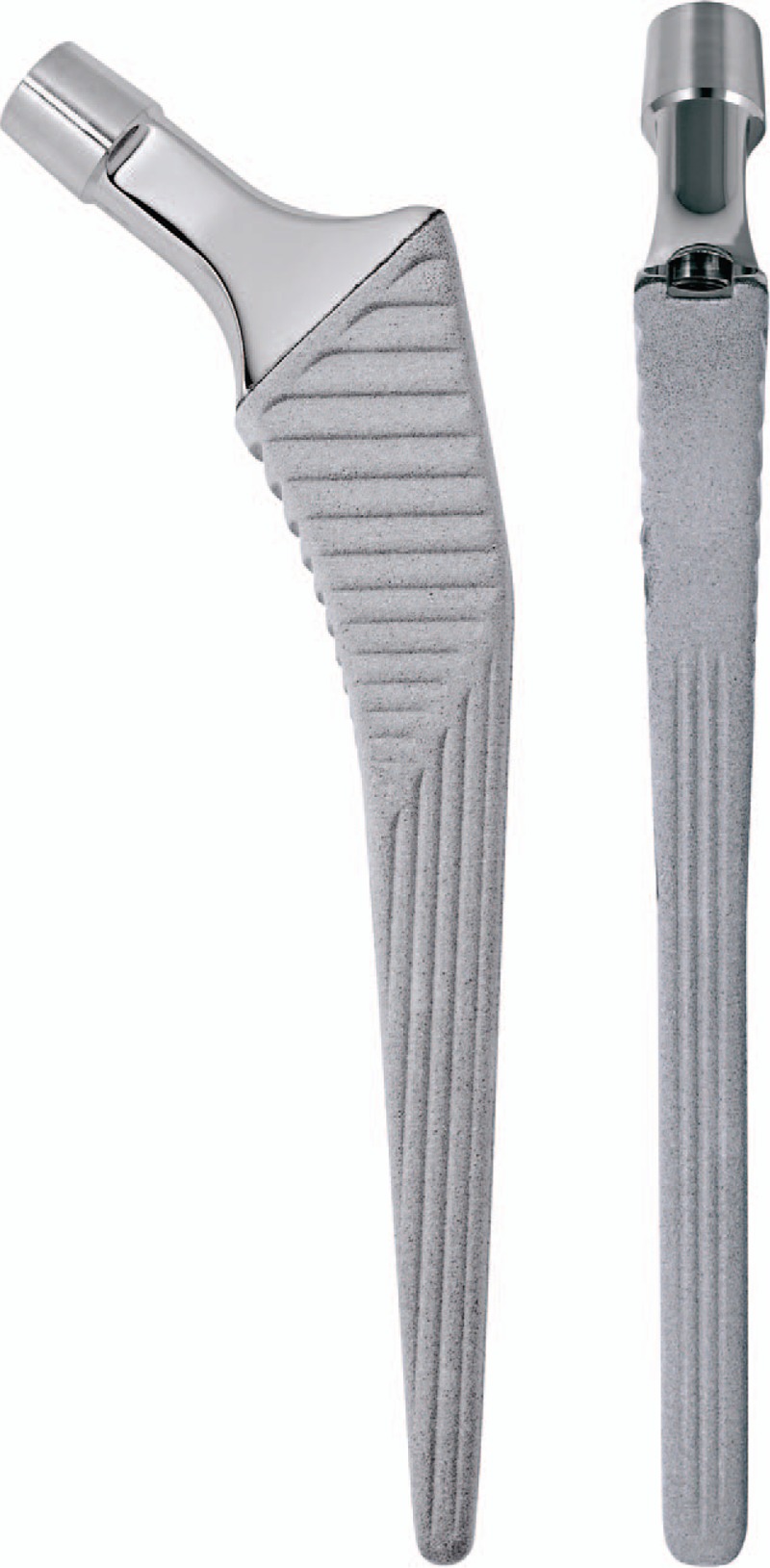
LCU cementless tapered femoral stem (LCU Hip system, Waldemar Link, Hamburg, Germany).

The Ribbed anatomic stem employs an anatomically s-shaped geometry, which permits the insertion of the greatest possible stem size into the medullary canal, thus achieving the aimed at form closure distally and proximally (Fig. 1). The broad prominent proximal ribs achieve a close fit to the supporting bone mass to provide the implant proximal fixation. While the LCU tapered stem employs a straight stem with tapered lateral shoulder, which is straight with a rectangular cross-section to give the implant proximal stability (Fig. 2). Both stems are made from forged titanium alloy (Ti6Al4V, Tilastan; Waldemar Link), the proximal portions of the Ribbed stem and the whole length of the LCU stem are provided with an osteoconductive calcium phosphate layer (HX Coating, about 15 μm thick). The detailed similarities and differences in the biomechanical properties between the Ribbed and LCU stems are listed in Table 1.

**Table 1:**
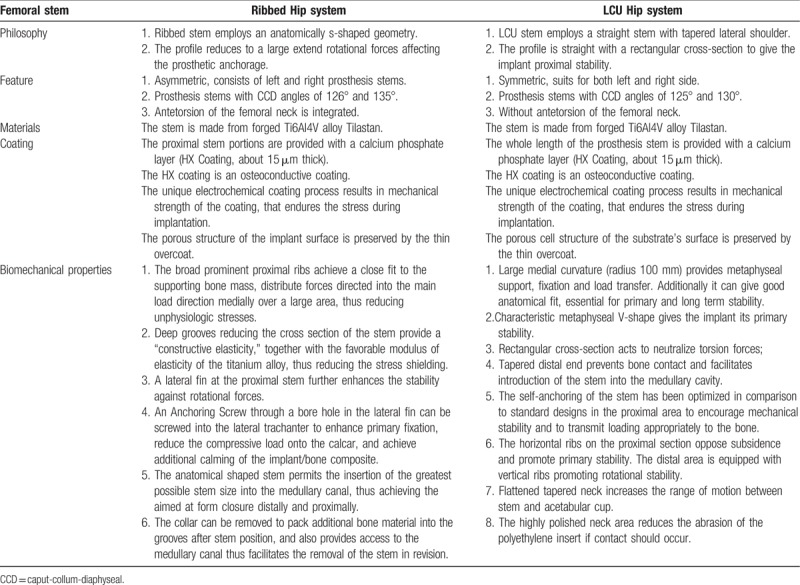
Comparison of anatomic (Ribbed Hip system) versus tapered (LCU Hip system) femoral stem design.

Both Trabeculae Oriented Pattern (TOP Acetabular Cup System; Waldemar Link) and TOP II (TOP II Acetabular Cup System; Waldemar Link) cementless hemispheric cup with a highly crosslinked polyethylene liner are used in both groups. Both metal-on-polyethylene and ceramic-on-polyethylene bearing surfaces are used; and majority of femoral head used in primary THA measuring 28 mm, larger diameter femoral head (32 mm) is seldom used.

### 2.5 Primary outcome measurement

**Figure 3:**
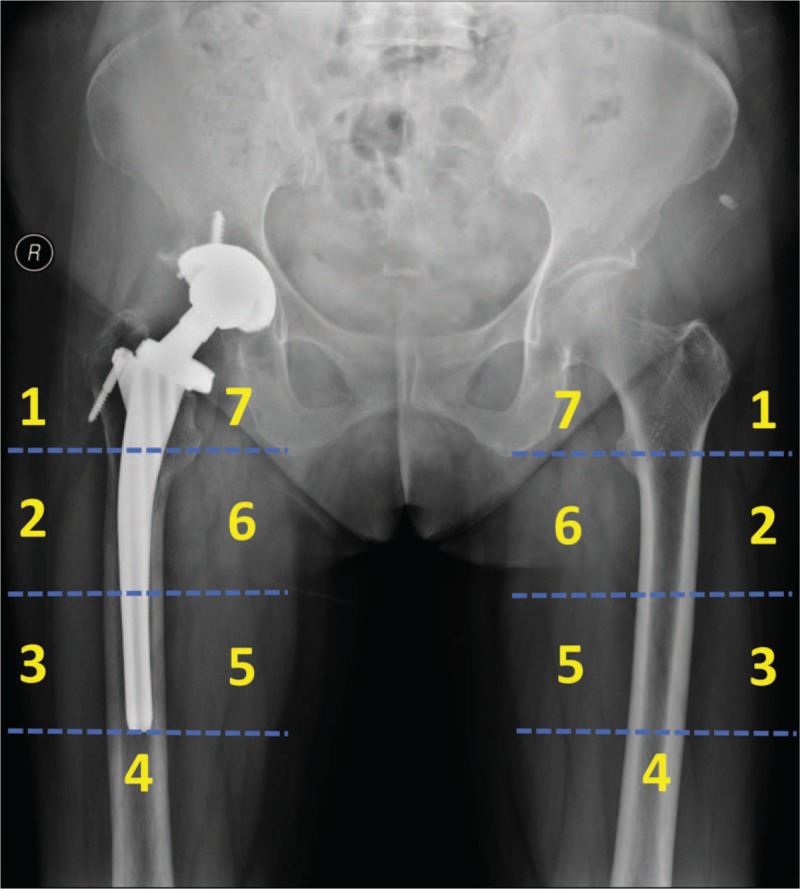
The defined Gruen zones of the periprosthetic side (Ribbed Hip system) and contralateral side.

**Figure 4:**
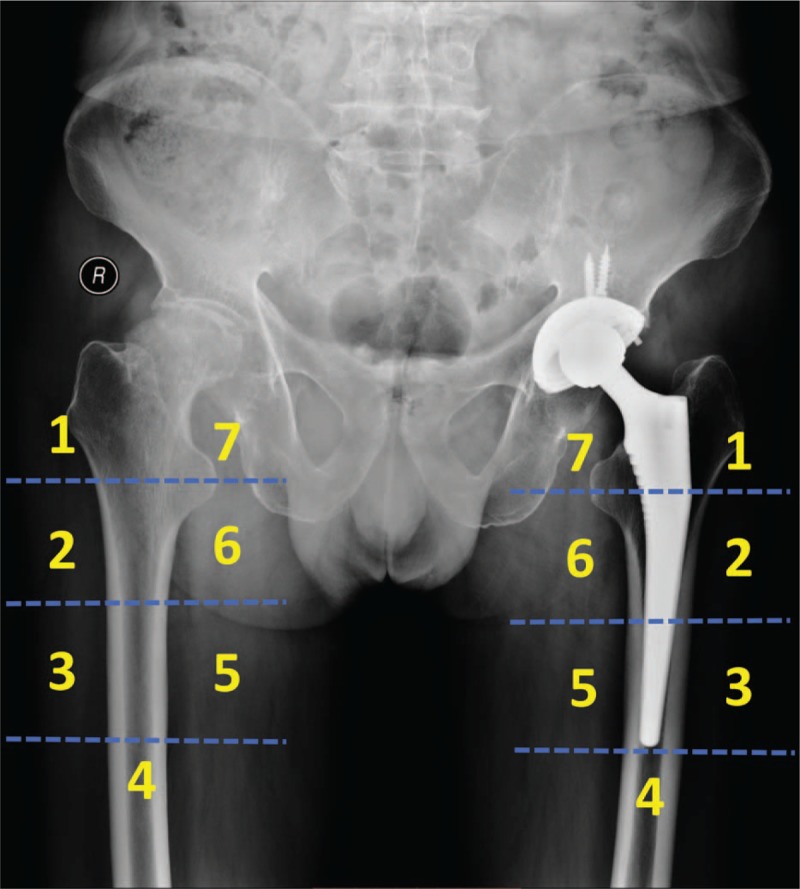
The defined Gruen zones of the periprosthetic side (LCU Hip system) and contralateral side.

The primary outcome in the present study is periprosthetic BMD changes, which will be accurately measured by dual-energy X-ray absorptiometry (DXA). Although innovative techniques as bone microarchitecture analysis (BMA) and high-resolution peripheral quantitative computed tomography (HRpQCT) have been developed to provide not merely BMD, but also more detailed information about the bone microarchitectural properties, DXA remains the most widely used and most thoroughly studied bone density measurement technology in research as well as in clinical practice.^[35,36]^ As a sensitive technique for determining BMD, DXA may actually detect the minor changes of periprosthetic BMD after THA, and facilitate the evaluation of periprosthetic bone remodeling.^[22,37]^ We will use the metal removal analysis algorithms of the Hologic Discovery instrument (Hologic Inc, Waltham, MA) to measure the periprosthetic BMD in 7 conventional regions of interest (ROIs) based on Gruen zones, which is the most often used protocol in evaluation of bone remodeling after the implantation of conventional femoral stems.^[38,39]^ BMD in each Gruen zone will be measured 2 days postoperatively on both prosthetic side and contralateral side, which will be taken as baseline value for an exact longitudinal comparison. Index-ROIs of the Ribbed and LCU stems will be defined based on the 1st postoperative pattern, this reference allows us to minimize the inter-time point variability and assuring measurement precision (Figs. 3 and 4).

### 2.6 Secondary outcome measurements

Consecutive conventional radiographs remain play an irreplaceable role in assessing and evaluating periprosthetic bone remodeling. Therefore, standard radiographs will be taken at each follow-up time point, which would be analyzed and measured by computer software to compare with the initial appearance on the radiographs taken immediately postoperatively. The cortical thickness will be measured in all 7 Gruen zones, and changes will be calculated to reflect the periprosthetic bone formation and simulated adaptation.^[38,40]^ Fitness of the distal stem within the isthmus of the femur will be evaluated on the anteroposterior radiograph, and according to the contact between the prosthesis and the femur, which would be classified as good (space <1 mm), fair (space 1–2 mm), or poor (space >2 mm).^[41]^ Femoral stem alignment in frontal plane will be measured and classified as neutral (within 3°), varus or valgus. The progress of radiolucent line and reactive line around the cementless femoral stem in the respective zones will be tracked.^[38,41,42]^ Periosteal bone reactions will be simply classified into incomplete or complete pedestal sign. Femoral component's subsidence will be measured and identified according to D’Antonio method.^[42]^ Biologic fixation and stability of the cementless implant will be evaluated using Engh grading scale (Table 2).^[43]^ The severity of heterotopic bone formation around the stem at each interval will be graded according to Brooker's classification.^[44]^ Besides, postoperative clinical outcomes including Harris hip score, Western Ontario and McMaster Universities Osteoarthritis Index (WOMAC) score will also assessed for functional evaluation.

**Table 2:**
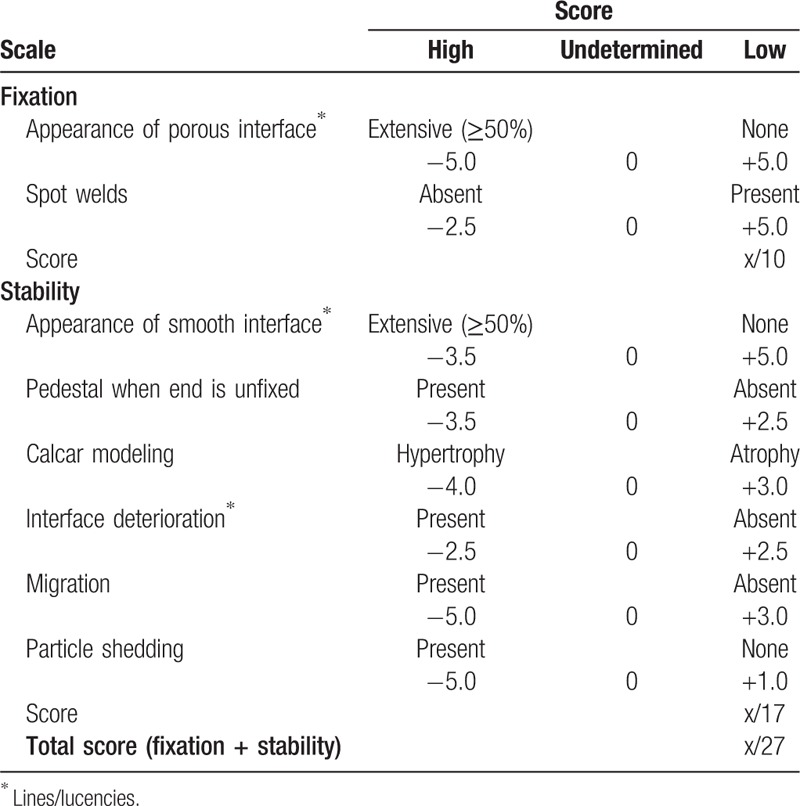
Engh grading scale for the radiologic evaluation of cementless total hip arthroplasty.

### 2.7 Sample size

The sample size was calculated based on the data obtained from our earlier cross-sectional study (http://www.chictr.org.cn, ChiCTR1800017750). We detected a mean difference in BMD of 0.1 g/cm^2^ with a standard deviation (SD) of 0.2 in patients received THA with the Ribbed stem. Based on these assumptions, setting an α error at 0.05 and the power level at 90%, additional compensate for possible dropout rate of 20%, a sample size of 50 patients in each group is required. We expect to recruit 100 patients within a period of 1 year based on our annual THA volume.

### 2.8 Statistical analysis

Frequencies and percentages will be estimated for qualitative data, and mean value ± SD will be calculated for quantitative data. To compare the periprosthetic BMD changes of Gruen zones between the Ribbed and LCU stems, statistical analysis will be performed using the Student *t* test for unmatched pairs to examine the significance of BMD changes of ROI 1–7, at each follow-up time point. And for further description of periprosthetic BMD changes, mean average BMD changes of ROI 1–7 will be presented as difference of both absolute and relative values (%) referred to the postoperative measurement. To minimize potential influential factors and better understanding the periprosthetic bone remodeling, we will also compare the periprosthetic BMD changes between the prosthetic side to the contralateral side by a paired *t* test for the Ribbed and LCU stems, respectively. For all analysis, a 2-tailed value of *P* < .05 is defined as statistically significant. Statistical analysis will be performed using the software SPSS version 22.0 (IBM Corporation, Armonk, NY).

### 2.9 Ethics and dissemination

The trial will be performed in compliance with the Declaration of Helsinki. Written informed consent will be obtained from all participating patients. Confidentiality of patients’ personal information will be protected. Each participant will be given a study identification number on enrolment, and data will be collected anonymously, ensuring that participants will not be identified through any data, transcripts, or publications. This study forms part of the authors’ graduation thesis, and will be assessed by the Chongqing Medical University. The findings of this study will be disseminated widely at national and international conferences, and will be published in peer reviewed, scientific journals.

## 3 Discussion

A variety of implant-, surgery-, and host-related factors have been delineated to explain the development of aseptic loosening and periprosthetic bone remodeling.^[45–47]^ The stem geometry is believed to play an important role in the load transfer to the femur, and consequently, in femoral remodeling and osseointegration.^[21,48]^ Dozens of previously published studies have reported the periprosthetic BMD changes following THA, but comparison of anatomic and tapered cementless femoral stems are rather limited.^[28,30,31,49–52]^ To the best of our knowledge, this will be the 1st study to compare the periprosthetic bone remodeling between the Ribbed and LCU stems. Although computer-simulation models like finite element analysis have been developed to calculate stress distribution, predict the extent of stress shielding, and long-term adaptive bone remodeling, the actual stress shielding and bone adaption in real-world working environments probably vary considerably, mainly because bone remodeling not only depend on mechanical factors but also more on biologic and physiologic ones.^[18,53–55]^ Therefore, periprosthetic BMD changes remain the optimal method available to reflect the long-term multi-factors involved bone remodeling.

The major limitation of this study is the technology limitation of DXA, which is unable to provide additional information about cortical and trabecular bone, and bone microarchitecture. The separate quantification of trabecular and cortical bone allows a better understanding of how bone is lost or formation in different regions, and future periprosthetic bone remodeling research using HRpQCT-technique-based BMA are warranted.^[35,56]^

In conclusion, this study will greatly contribute to a better understanding of the stem geometry and periprosthetic bone remodeling. The findings of this study would be valuable for improving the design of implants, and will act as a guide for the revolutionary of prosthesis designs.

## Author contributions

**Conceived and designed the study:** Xiang-Dong Wu, and Wei Huang;

**Drafted the study protocol:** Xiang-Dong Wu, Yu Chen, Zhang-Yu Wang, Yu-Jian Li, Zheng-Lin Zhu, Yu-Zhang Tao, Wei Huang;

**Final approval of the version to be published:** Xiang-Dong Wu, Yu Chen, Zhang-Yu Wang, Yu-Jian Li, Zheng-Lin Zhu, Yu-Zhang Tao, Hong Chen, Qiang Cheng, Wei Huang.

**Responsible for data collection, analysis and interpretation:** Xiang-Dong Wu, Yu Chen, Zhang-Yu Wang, Yu-Jian Li, Zheng-Lin Zhu, Yu-Zhang Tao, Hong Chen, Qiang Cheng, Wei Huang;

**Responsible for managing for the project and conducting formal analysis:** Xiang-Dong Wu, Yu Chen, Zhang-Yu Wang, Yu-Jian Li, Zheng-Lin Zhu, Yu-Zhang Tao, Hong Chen, Qiang Cheng, Wei Huang;

**Responsible for study implementation:** Xiang-Dong Wu, Yu Chen, Zhang-Yu Wang, Yu-Jian Li, Zheng-Lin Zhu, Yu-Zhang Tao, Hong Chen, Qiang Cheng, Wei Huang;

**Reviewed and revised the study protocol:** Xiang-Dong Wu, Yu Chen, Hong Chen, Qiang Cheng, Wei Huang;

**Conceptualization:** Xiang-Dong Wu, Wei Huang.

**Data curation:** Xiang-Dong Wu, Yu Chen, Zhang-Yu Wang, Yu-Jian Li, Zheng-Lin Zhu, Yu-Zhang Tao, Hong Chen, Qiang Cheng, Wei Huang.

**Formal analysis:** Xiang-Dong Wu, Yu Chen, Zhang-Yu Wang, Yu-Jian Li, Zheng-Lin Zhu, Yu-Zhang Tao, Hong Chen, Qiang Cheng, Wei Huang.

**Funding acquisition:** Wei Huang.

**Investigation:** Xiang-Dong Wu, Yu Chen, Zhang-Yu Wang, Yu-Jian Li, Zheng-Lin Zhu, Yu-Zhang Tao, Hong Chen, Qiang Cheng, Wei Huang.

**Methodology:** Xiang-Dong Wu, Yu Chen, Zhang-Yu Wang, Yu-Jian Li, Hong Chen, Qiang Cheng, Wei Huang.

**Project administration:** Xiang-Dong Wu, Zhang-Yu Wang, Hong Chen, Qiang Cheng, Wei Huang.

**Resources:** Xiang-Dong Wu, Yu Chen, Zheng-Lin Zhu, Yu-Zhang Tao, Hong Chen, Wei Huang.

**Software:** Xiang-Dong Wu, Yu Chen, Zhang-Yu Wang, Zheng-Lin Zhu, Yu-Zhang Tao, Hong Chen, Qiang Cheng, Wei Huang.

**Supervision:** Wei Huang.

**Validation:** Xiang-Dong Wu, Hong Chen, Qiang Cheng, Wei Huang.

**Visualization:** Xiang-Dong Wu, Yu Chen, Yu-Zhang Tao, Qiang Cheng, Wei Huang.

**Writing – original draft:** Xiang-Dong Wu, Yu Chen, Zhang-Yu Wang, Yu-Jian Li, Zheng-Lin Zhu, Yu-Zhang Tao.

**Writing – review & editing:** Xiang-Dong Wu, Yu Chen, Hong Chen, Qiang Cheng, Wei Huang.

Wei Huang orcid: 0000-0002-8894-0982
